# Chronic Ventriculitis Caused by Cryptococcus neoformans: A Rare Presentation

**DOI:** 10.7759/cureus.48926

**Published:** 2023-11-16

**Authors:** Ashwin Ragupathi, Marian Rodriguez-Carbo, Sydney Weisman, Cuc Mai

**Affiliations:** 1 Internal Medicine, University of South Florida Morsani College of Medicine, Tampa, USA

**Keywords:** intermittent headaches, non-obstructive hydrocephalus, hiv, chronic ventriculitis, cryptococcus neoformans

## Abstract

Cryptococcus neoformans is a fungus notorious for invading the central nervous system. while Cryptococcus is known to cause meningitis, encephalitis, and meningoencephalitis in immunocompromised patients, especially those with AIDS (CD4 <100), and found to be rapidly fatal, instances of ventricular involvement with chronic sequelae are exceedingly rare. Typical presentations of cryptococcal meningitis involve headache, altered mental status, nuchal rigidity, and vomiting. We report a case of a 58-year-old HIV-positive male who presented with intermittent headaches and changes in gait. The MRI revealed ventriculomegaly and advanced chronic sequela of prior ventriculitis with serum and CSF cryptococcal antigen being positive. The treatment of cryptococcal chronic ventriculitis requires a multidisciplinary approach involving internal medicine, neurosurgery, neurology, and infectious diseases. However, this patient’s CSF had no pleocytosis and had very high protein, which is a poor prognostic indicator for this disease and could have been prevented with the prompt recognition of the condition before it had progressed to the chronic stage. We recommend that clinicians maintain a high index of suspicion for opportunistic infections, such as cryptococcal meningitis, in any patient with HIV regardless of typical clinical findings.

## Introduction

Cryptococcus neoformans is a fungus transmitted through the inhalation of spores that colonize the lungs. It disseminates hematogenously through blood, to the blood-brain barrier, thereby causing infection in the central nervous system [1]. While it can infect any individual, it is only in patients who are immunocompromised, such as those with AIDS, transplant patients, and patients with other immunodeficiency syndromes, that the disease tends to be fatal, causing about 100,000 HIV-related deaths per year worldwide [2]. While cryptococcus can infect the central nervous system, causing meningitis, the incidence of ventricular involvement with a chronic sequela tends to be very rare. We report a case of chronic ventriculitis caused by cryptococcus; we also engage in a discussion of the case history, diagnostic modalities, treatment complications, and prevention.

## Case presentation

A 58-year-old male with a medical history of HIV for two years [off highly active antiretroviral therapy (HAART) for two months] and squamous cell carcinoma (SCC) of the right base of the tongue/tonsil with lymphovascular invasion presented to the emergency department with complaints of intermittent headaches, dizziness, progressive memory loss, and changes in his gait. Physical examination revealed oral candidiasis, temporal wasting, and a solid mass in the lower right mandible. A neurological examination showed that the patient was conscious and oriented but was not able to recall when the symptoms had started or his previous admission details. The power on the legs was 4/5 on the right and 5/5 on the left; other neurological examinations such as those involving cranial nerves, sensory function, and reflexes were unremarkable. Ophthalmological examination showed no evidence of papilledema. There was no evidence of nuchal rigidity and no complaints of vomiting. The patient's complete blood count (Table 1) and complete metabolic profile (Table 2) were normal and his absolute CD4 cells were 334 (Table 3).

**Table 1:** Complete blood count

Parameters	Values
White blood cells	6.06
Red blood cells	4.04
Hemoglobin	9.8
Hematocrit	32.3
Platelet	548

**Table 2:** Complete metabolic profile

Parameters	Values
Sodium	137
Potassium	3.6
Chloride	106
Carbon dioxide	21
Blood urea nitrogen	5
Glucose	72
Creatinine, blood	0.8
Calcium	9.1
Aspartate aminotransferase	20
Alanine aminotransferase	14
Total bilirubin	0.2
Alkaline phosphatase	80
Total protein	7.6
Albumin	2.9
Anion gap	10
Blood urea nitrogen/creatinine ratio	6
Globulin	5
Albumin/globulin ratio	1
Glomerular filtration rate	103
Thyroid-stimulating hormone	0.73

**Table 3:** CD4/CD8 count and ratio profile

Parameters	Values
Absolute lymphocyte count, % CD4 (T cells)	1391, 24%
Absolute CD4	334
% CD8 (T cells)	52%
Absolute CD8	723
T4:T8	0.46

**Figure 1:**
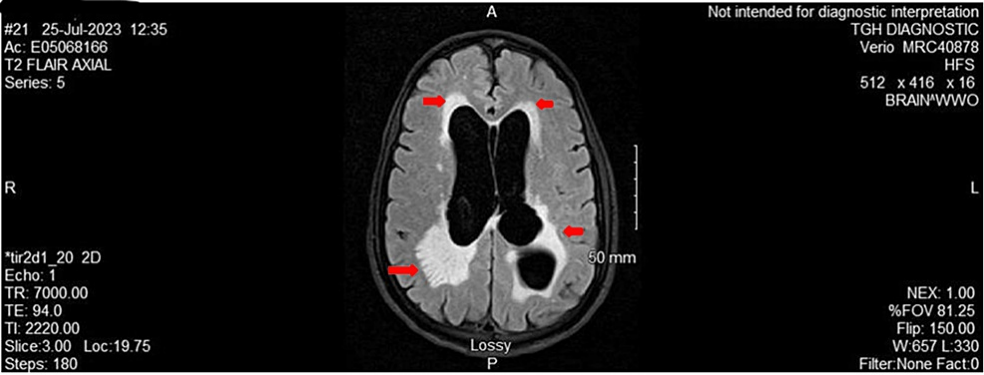
MRI brain: T2 FLAIR axial The image shows bilateral periventricular white matter hyperintensities FLAIR: fluid attenuated inversion recovery; MRI: magnetic resonance imaging

**Figure 2:**
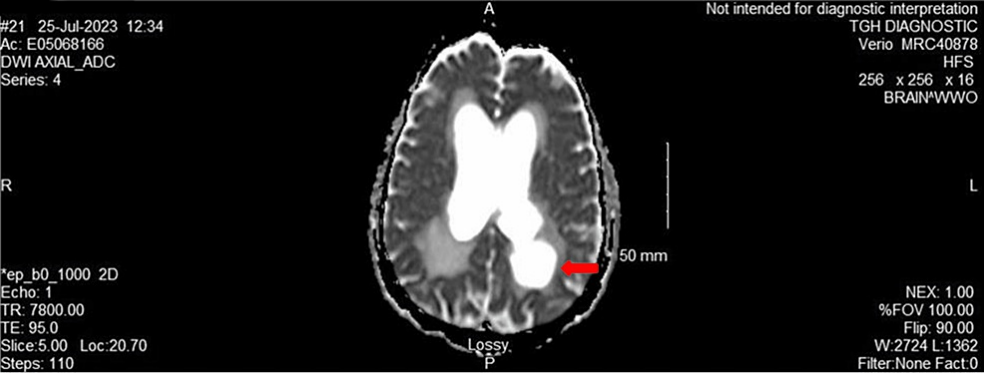
MRI brain: DWI axial ADC The image shows ex vacuo dilatation of the occipital horn of the left lateral ventricle ADC: apparent diffusion coefficient; DWI: diffusion-weighted imaging; MRI: magnetic resonance imaging

Given the patient's neurological symptoms, we performed a CT brain, which showed communicating hydrocephalus and transependymal flow of CSF, potentially in the setting of ventriculitis. An MRI was obtained for a more detailed image, and it revealed ventriculomegaly of all four ventricles with irregularity of ventricular contour and complex lobulated cystic changes in the midline. The ventricles also had extensive scarring and adhesions throughout (Figures 1, 2). These findings were consistent with advanced chronic sequela of prior ventriculitis. Given his risk factors for opportunistic infections, a serum Cryptococcus antigen, TB QuantiFERON, and blood cultures were obtained. Cryptococcal antigen returned positive with a titer of 1:80 (Table 4). His CSF analysis showed xanthochromia and an RBC count of 1000, which would be expected in HSV encephalitis, but HSV 1 and 2 were negative and CSF came back positive for cryptococcal antigen (Table 5). Neurocritical care was consulted and a temporary external ventricular drain (EVD) was placed (noted OP <10 at the time of EVD placement), which improved the patient's headache. He had disseminated cryptococcosis with chronic cryptococcal meningitis as the CSF culture came back positive. The CSF had no pleocytosis and had very high protein, which are poor prognostic indicators for this disease. Induction therapy for cryptococcal meningitis with liposomal amphotericin B 5 mg/kg and flucytosine 1500 mg q6h was initiated.

**Table 4:** Microbiology profile CSF: cerebrospinal fluid; DNA: deoxyribonucleic acid

Microbes	Detection
Cryptococcal antigen, CSF titer	Positive (1:5)
Cryptococcal antigen, serum titer	Positive (1:80)
Cytomegalovirus DNA CSF	Not detected
Enterovirus DNA CSF	Not detected
Haemophilus influenza DNA CSF	Not detected
Herpes simplex virus 1 DNA CSF	Not detected
Herpes simplex virus 2 DNA CSF	Not detected
JC polyomavirus DNA CSF	Not detected
Listeria monocytogenes DNA CSF	Not detected
Neisseria meningitis DNA CSF	Not detected
Paraechovirus RNA CSF	Not detected
Streptococcus agalactiae DNA CSF	Not detected
Streptococcus pneumoniae DNA CSF	Not detected
Streptococcus group A DNA	Not detected
Leukemia/lymphoma PNL	Not detected
Varicella-zoster DNA CSF	Not detected
Human herpes virus-6 DNA CSF	Not detected
Escherichia coli DNA CSF	Not detected

**Table 5:** CSF analysis CSF: cerebrospinal fluid; RBC: red blood cells

Parameters	Values
CSF color	Xanthochromia
CSF character	Clear
CSF RBC	1000
CSF neutrophils	7
CSF lymphocytes	63
CSF monocytes	30
CSF glucose	64
CSF total protein	123

## Discussion

HIV is a retrovirus that targets CD4 T cells of the body. People living with HIV can have a normal lifespan with no HIV-related complications if they have access to and are adherent to antiretroviral therapy. However, people living with untreated HIV, especially with a CD4 count below 200, are vulnerable to fatal opportunistic infections. This is also true for other immunocompromised patient populations, such as transplant patients requiring immunosuppressive therapy [2]. Cryptococcus neoformans is an encapsulated yeast that can be found in aged pigeon droppings and causes mild infections, such as airway colonization or asymptomatic ones in laboratory workers, or severe infections like meningitis or disseminated disease pneumonia or meningoencephalitis [2]. Meningoencephalitis commonly presents with severe headache, vomiting, blurring of vision, nuchal rigidity, and altered mental status. While individuals with HIV and a CD4 count below 100 are at significant risk of cryptococcal antigenemia and have an increased likelihood of progressing to cryptococcal meningitis, it is important to note that the possibility of cryptococcal meningitis should not be ruled out in patients with a CD4 count above 100, as was the case with our patient.

Laboratory exams usually involve serum antigen testing, CSF antigen testing, CSF culture, CSF India ink staining, CSF fluid analysis, and increased opening pressure. Lumbar puncture (LP) should be performed only after a CT or MRI is obtained to exclude a mass lesion, which, if present, can lead to a brain herniation following an LP. As per CSF analysis, the increased opening pressure would be >25 mmH_2_O and the CSF analysis would have decreased glucose, increased protein, and a lymphocyte predominance. Typical findings in an MRI would include leptomeningeal enhancement and pachymeningeal enhancement on T1 C+ (Gd). Classically, there is a “soap bubble” appearance when dilated perivascular space coalesces into gelatinous pseudocysts [3].

In our case, the patient had headaches and changes in gait, which led us to think he might have had an embolic stroke or a metastasis from the SCC. Imaging showed chronic ventriculitis, which was atypical for Cryptococcus, but we maintained a high index of suspicion for Cryptococcus because the patient was infected with HIV. Hence, we did a serum antigen test. The serum antigen test came back positive and we proceeded with CSF studies for Cryptococcus. The patient's CSF culture for cryptococcus came back positive and CSF analysis revealed poor pleocytosis and high protein due to which the patient had a poor prognosis. Neurocritical care was consulted and a temporary EVD was placed to alleviate his symptoms, which relieved his headache. Induction therapy with liposomal amphotericin B 5 mg/kg and flucytosine 1500 mg q6h was started. Patients with chronic cryptococcal ventriculitis require a multidisciplinary care team to treat the disease adequately. Patients may present with chronically relapsing cryptococcal meningitis caused by Cryptococcus neoformans if there is inadequate treatment or if there is a diagnostic delay [4]. According to a study by Roy and Chiller, the cryptococcal antigen would be present in the blood several weeks before the onset of symptoms a simple point-of-care testing for cryptococcal antigen at the time of entry into the hospital for patients with a known history of HIV could prevent fatalities [5]. If patients test positive for cryptococcal antigen, they should be followed up regularly, and if they develop symptoms of meningitis, we should perform LP for further analysis.

## Conclusions

Cryptococcal meningitis can occasionally manifest without the hallmark symptoms typically associated with meningitis, such as vomiting, blurred vision, nuchal rigidity, and altered mental status. In some cases, the presence of concurrent medical conditions, such as SCC, can obscure the diagnosis. Consequently, it is crucial to consider the possibility of cryptococcal meningitis in patients with predisposing factors for opportunistic infections, particularly those with untreated HIV, even if their CD4 cell counts remain above 100. Failure to promptly recognize and address cryptococcal meningitis in such individuals can lead to severe complications, including disseminated infection, obstructive hydrocephalus, seizures, and, tragically, fatal outcomes. Therefore, healthcare professionals should maintain a high index of suspicion and perform thorough evaluations to ensure early detection and appropriate management in these high-risk populations.
